# Early experience with ovation endograft system in abdominal aortic disease

**DOI:** 10.1186/1749-8090-9-48

**Published:** 2014-03-12

**Authors:** Giovanni Nano, Daniela Mazzaccaro, Silvia Stegher, Maria Teresa Occhiuto, Giovanni Malacrida, Domenico G Tealdi, Antonino Alberti, Pietro Volpe

**Affiliations:** 1 First Unit of Vascular Surgery, IRCCS Policlinico San Donato, University of Milan, Piazza E. Malan, 1, 20097 San Donato Milanese, MI, Italy; 2 Department of Vascular Surgery, Riuniti Hospital of Reggio Calabria, Reggio Calabria, Italy

**Keywords:** Abdominal aortic aneurysm, Endovascular aneurysm repair, Ovation, Stent graft

## Abstract

**Objective:**

We describe our initial experience with the use of the TriVascular Ovation endograft system for the treatment of abdominal aortic aneurysms (AAA).

**Methods:**

We retrospectively reviewed data from patients treated for AAA using the Ovation endograft at two institutions from January 2011 to September 2012. Main outcomes included primary success, survival, complications, and device-related events. The mean follow-up period was 10 months (range 1–22 months).

**Results:**

Thirty-seven patients (male: 95%, mean age: 76 yr) were treated for AAA (mean diameter: 54 mm) with the Ovation endograft. Local or regional anesthesia was used in 86.5% of cases. Percutaneous access was utilized in 73% of cases. Primary success was 89.2% (33/37). Four adjunctive procedures were required including two distal extensions (type 1b endoleak and iliac limb disconnection resulting in type III endoleak) and two bypass surgeries (limb graft occlusion and gate cannulation failure). No deaths or major complications were reported during the procedure or in follow-up. No type I, III, or IV endoleak, AAA enlargement, AAA rupture, stent fracture, migration, or endovascular or surgical reintervention were reported during the follow-up period. Type II endoleak was observed in two patients. Asymptomatic narrowing of both iliac limbs was observed in one patient at 6 months.

**Conclusions:**

Our initial experience with the Ovation endograft demonstrated encouraging results in patients with AAA.

## Background

The first endovascular repair of an abdominal aortic aneurysm (AAA) was performed in 1990 by Dr. Parodi [1], who implanted a large straight endograft into the abdominal aorta. Limitations of the use of a similar device soon became evident and new self-expandable materials and bifurcated stent-grafts, which had a smaller caliber than previous generations, rapidly became available. The latest generation of ultra-low profile endografts currently allows the treatment of AAAs in patients who have previously been excluded because of challenging aortic anatomies and small access vessels.

However, some have expressed concern that reducing the profile of a stent graft may compromise sealing. The search for an endograft with a smaller profile with no compromise on performance and durability of the device remains a therapeutic challenge. After having received the CE Mark on September 17th 2010, the low-profile TriVascular Ovation system (14 F) has been recently introduced also to our institutions for use in selected patients with relatively challenging necks or smaller iliac vessels. The purpose of this study was to retrospectively review our early experience with this novel stent graft.

## Methods

### Ethics

The research described in this manuscript was approved by the Institutional Review Board at each respective institution and was conducted per the guidance set forth in the Declaration of Helsinki.

### Patients

We retrospectively reviewed data of all patients who were treated for AAA using the Ovation endograft system from January 2011 to September 2012 in our two centres.

Patients who meet specific anatomic criteria of both proximal and distal landing zone (non-aneurysmal proximal aortic neck length of at least 7 mm, proximal neck luminal diameter between 16 and 30 mm, non-aneurysmal distal iliac artery length of at least 10 mm, distal iliac luminal diameters between 8 and 20 mm, possibility to preserve patency in at least one hypogastric artery, distance from the most distal renal artery to most superior internal iliac artery measurement of at least 13 cm) were considered suitable for the implantation of the Ovation Abdominal Stent Graft.

One patient was treated outside the IFU of the Ovation Stent Graft, with a pre-planned “chimney-technique” because of AAA involving both renal arteries. Due to small iliac access, however, it was not possible to treat the patient using a different type of endograft.

Patients with a dissecting or acutely ruptured aneurysm were excluded, as well as patients with history of connective tissue disease (e.g., Marfan's or Ehler's-Danlos syndrome) or with a known allergy or intolerance to polytetrafluorethylene (PTFE), PEG-based polymers, fluorinated ethylene propylene (FEP) or nitinol.

After treatment all patients were discharged on antiplatelet therapy (either ASA 100 mg daily or Clopidogrel 75 mg daily all lifelong) unless they were already on anticoagulant therapy for preoperative comorbidities.

### Ovation stent graft

**Figure 1:**
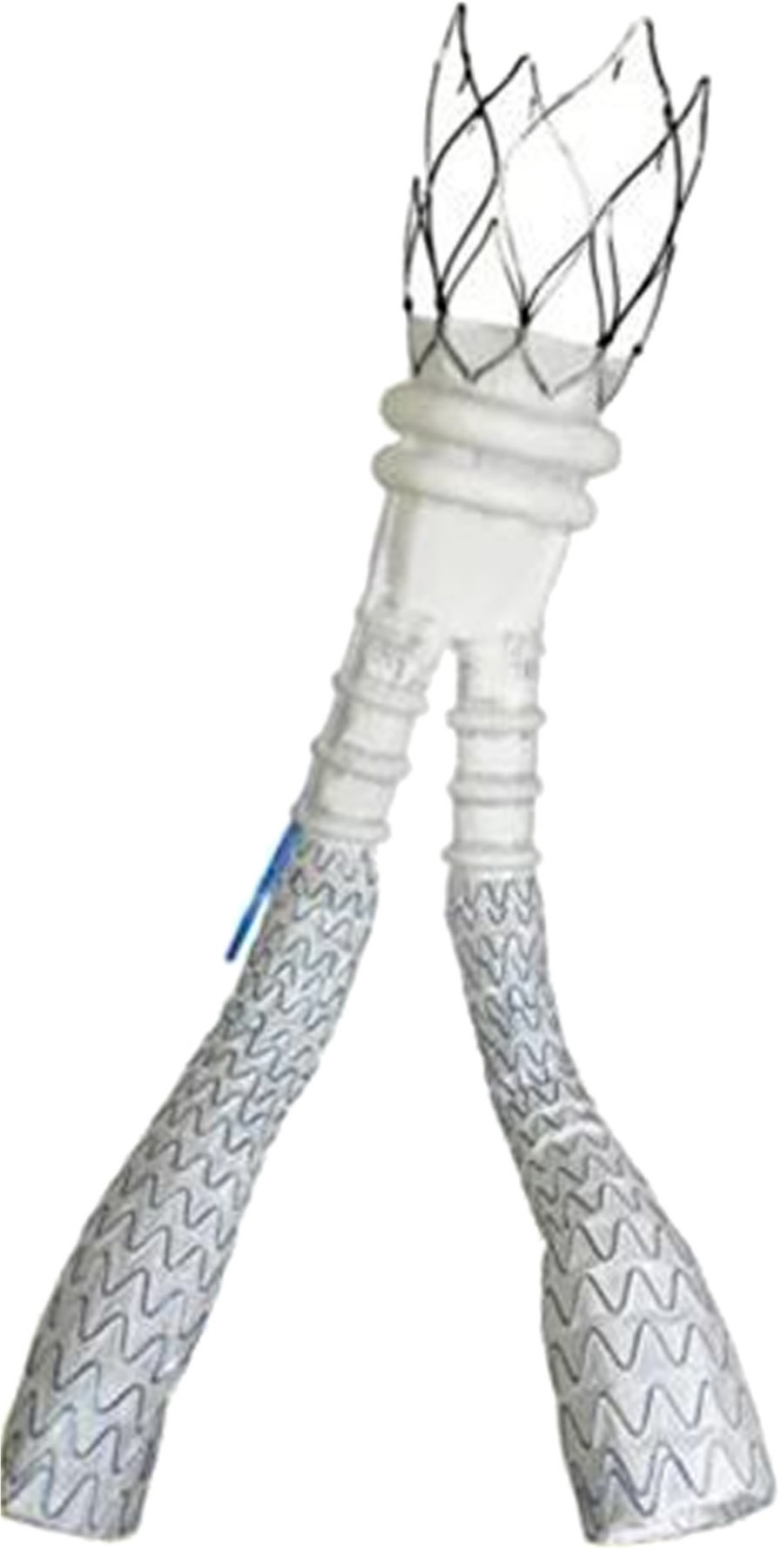
The Ovation Trivascular graft.

The Ovation Abdominal Stent Graft (TriVascular, Inc., Santa Rosa, CA, USA) is a modular two-docking limb device system with the aortic body delivered via a flexible hydrophilic-coated 14 Fr OD catheter, as well as the contralateral limb. The aortic body is comprised of a low permeability PTFE graft and a suprarenal nitinol stent with integral anchors to achieve active fixation to the aortic wall. The iliac limbs are made of a low permeability PTFE graft with an external scaffold of nitinol stents. The Ovation system has ring-shaped channels (Figure 1) that are injected with a polyethylene glycol [PEG]-based polymer, which expands the endograft against the aorta to create a proximal seal.

The polymer is radiopaque, allowing the filling of the graft to be observed under fluoroscopy. Once the main body of the graft is positioned and opened, the polymer is prepared on the operating table, and mixed in a syringe that will be joined to the graft introducer. The syringe itself is joined to an autoinjector which pushes the polymer through the ring-shaped channels in 20 minutes, during which it is possible to complete the placement of the contralateral iliac extension. When the time of the polymer’s filling is elapsed, the syringe and the autoinjector are removed together and it is possible to place the eventual ipsilateral iliac extension.

Because of the ultra-low profile, the Ovation system can be used in patients with iliac or femoral artery access of less than 7 mm. The Ovation device is contraindicated in patients who have allergies to the device materials, as well as in patients who are unable to undergo the necessary preoperative and postoperative imaging studies.

### Outcomes

Patient records were reviewed for demographics, medical history, and aortoiliac morphology via computed tomography angiography. Procedural data included fluoroscopy time, operation time, amount of procedural contrast medium, blood loss, and complications. Follow-up data were obtained from outpatient visits and CT scan or duplex scans. Imaging was performed every 3 months during the first year after the operation and annually thereafter using mainly duplex scans. A CT scan was regularly performed at 1 year and in any case of inconclusive duplex scan (i.e. hostile abdomen) or finding of sac enlargement/graft occlusion/stenosis/endoleaks at duplex scan. Outcomes were analyzed to evaluate primary success, survival, complications, and device-related events. Primary success was defined as successful access, delivery, and implant of the endograft with absence of surgical conversion, mortality, type I or III endoleak, or graft limb obstruction.

### Data analysis

Statistical analysis was performed using JMP® 5.1.2 (SAS Institute, Inc., Cary, NC, USA). Continuous variables are reported as mean ± SD and categorical variables are presented as n (%). Logistic regression using the Wald statistic was performed to identify predictors of adjunctive procedures. P values < 0.05 were considered statistically significant.

## Results

### Baseline patient characteristics

Thirty-seven patients (35 males, mean age 76 years) with AAA (mean 54 mm diameter) were treated with the Ovation endograft. Primary AAA etiology was atherosclerotic and degenerative (n = 35) while 2 patients presented with aortic pseudoaneurysm of proximal anastomosis from previous AAA open surgery. The most common patient comorbidities included current or previous smoking (51%), chronic obstructive pulmonary disease (35%), and previous coronary artery bypass grafting (32%). No patient presented with severe renal failure. Twenty nine percent of treated aortic necks were shorter than 15 mm and 15% were less than 10 mm in length. Twenty percent of patients treated had an access vessel less than 7 mm in diameter, including a minimum diameter of 4.7 mm (Table 1).

**Table 1:** Patients’ characteristics and anatomical data

	**n = 37**
**Male Sex**	35 (94.6%)
**Mean age, years (Range)**	75.5 (60–90)
**Aetiology**	
Atherosclerosis/Degeneration	35 (94.6%)
PSA	2 (5.4%)
**Comorbidities**	
Current or previous smoke	19 (51.3%)
COPD	13 (35.1%)
Previous CABG	12 (32.4%)
Hypertension	8 (21.6%)
Dislipidemia	6 (16.2%)
Diabetes	5 (13.5%)
Obesity	5 (13.5%)
Neoplasm	2 (5.4%)
Stroke	1 (2.7%)
**Anatomical data (Mean ± SD), mm**	
Proximal aortic neck diameter	25.9 ± 0.8
Proximal aortic neck lenght	18 ± 2.1
Proximal aortic neck angulation	52.2° ± 4.2°
Distal aortic neck diameter	27.7 ± 0.4
Distal aortic neck lenght	24 ± 1.2
Proximal RCIA	12.3 ± 0.2
Distal RCIA	14.2 ± 0.5
Proximal LCIA	14.2 ± 0.4
Distal LCIA	13.9 ± 0.7
Smaller EIA	8.1 ± 0.7
Sac diameter (range)	5.4 (5.0-8.5)
Aortic length	82 (58–122)

PSA = Pseudoaneurysm.

COPD = Chronic Obstructive Pulmonary Disease.

CABG = Coronary Artery Bypass Grafting.

RCIA = Right Common Iliac Artery.

LCIA = Left Common Iliac Artery.

EIA = External Iliac Artery.

### Procedural data

Intraprocedural data are described in Table 2. All procedures were performed by vascular surgeons in the operating theatre. Most (86.5%) procedures were performed using either local or regional anesthesia. Endovascular repair was performed via percutaneous bilateral femoral access in 27 patients, while in the remaining 10 cases bilateral surgical exposure of both common femoral arteries was necessary.

**Table 2:** Intraprocedural and In-hospital data (Mean ± SD)

	**n = 37**
**Anesthesia**	
Regional	25 (67.6%)
Local + conscious sedation	7 (18.9%)
General	5 (13.5%)
**Vascular access**	
Percutaneous femoral	27 (73%)
Percutaneous brachial (additional)	2 (5.4%)
Surgical femoral	10 (27%)
**Time of operation (min)**	43.1 ± 3.2
**Amount of contrast (cc)**	28.3 ± 2.5
**Fluoroscopy time (min)**	11.3 ± 1.5
**Blood loss (cc)**	110 ± 16
**Primary success**	33 (89.2%)
Adjunctive procedure*	4 (10.8%)
**Length of stay (days)**	3.6 ± 0.9
**30-day results**	
Death	0 (0%)
Major adverse events	0 (0%)
Endoleak	0 (0%)
**Long-term results**	
Death	0 (0%)
Major adverse events	0 (0%)
Endoleak type II	2 (5.4%)
Asintomatic narrowing of iliac limbs	1 (2.8%)

Thirty-day and long-term results.

*two distal extensions (type 1b endoleak and iliac limb disconnection resulting in type III endoleak) and two bypass surgeries (limb graft occlusion and gate cannulation failure).

An additional percutaneous brachial access was performed in 2 patients in which a “Chimney Technique” was used. In one of these cases, the AAA involved the origin of both renal arteries while in the remaining case the chimney graft was used as a bail-out technique, allowing a safe proximal landing zone without covering the right renal artery. In both patients, a Viabahn® covered stent was advanced antegrade through brachial access. The proximal rings of the Ovation graft were inflated while a balloon was kept inflated within the covered stent.

All grafts were oversized less than 20%; in both cases in which a “Chimney Technique” was used, the graft had a 10% oversize.

Closure of the access site was obtained with manual compression bilaterally in 2 out of 27 cases of percutaneous femoral access. A vascular closure device was used in the remaining cases of percutaneous femoral access.

Primary success was achieved in 33 cases (89.2%). Four patients required adjunctive procedures during the operation. In one case, given the twist of the device, which opened incompletely at the gate, we were not able to canulate the contralateral leg. Therefore, although a good proximal seal had been obtained, a conical stent graft (Talent, Medtronic) was placed, with an occluder at the level of the left common iliac artery, and a femoro-femoral right-to-left bypass was performed. In the second case, intraoperative graft occlusion of the right leg required an additional femoro-femoral left-to-right bypass. In the third case, the device failed despite proper deployment with enough overlap. Disconnection between the main body and the left leg occurred, causing an intraprocedural type III endoleak which was immediately corrected with a second iliac extension. In the fourth case, a type Ib endoleak at the end of the procedure required the placement of a distal iliac extension.

No patient required a blood transfusion or ICU stay. No access-related complication or post-implantation syndrome was reported. The risk of adjunctive procedures was higher in patients with smaller proximal neck diameter (OR 12, 95% CI 2.36-24.17, P = .02) (Table 3), but it should be noted that these complications usually are not correlated to proximal neck diameter.

**Table 3:** Logistic regression predicting risk of adjunctive procedures from aortoiliac characteristics

	***ODDS RATIO***	***95%CI***	***P***
***Proximal aortic neck diameter***	**12**	**2.36 - 24.17**	**.02**
***Proximal aortic neck length***	0.82	0.21 – 1.80	.12
***Distal aortic neck diameter***	1.26	0.36 – 4.82	.08
***Distal aortic neck lenght***	2	0.73 – 5.16	.21
***Proximal RCIA***	0.42	0.01 – 2.23	.32
***Distal RCIA***	0.99	0.67 – 4.11	.69
***Proximal LCIA***	2.18	0.87 – 3.92	.23
***Distal LCIA diameter***	1.6	0.09 – 3.2	.40
***Sac diameter***	2.21	0.58 – 3.64	.28
***Aortic length***	1.12	0.77 – 2.14	.12
**EIA diameter**	0.90	0.42 – 1.23	.38

Nominal logistic fit, Wald effect test 1 DF. Significant P value in bold.

CI = Confidence Interval.

RCIA = Right Common Iliac Artery.

LCIA = Left Common Iliac Artery.

EIA = External Iliac Artery.

### Safety and effectiveness outcomes

**Figure 2:**
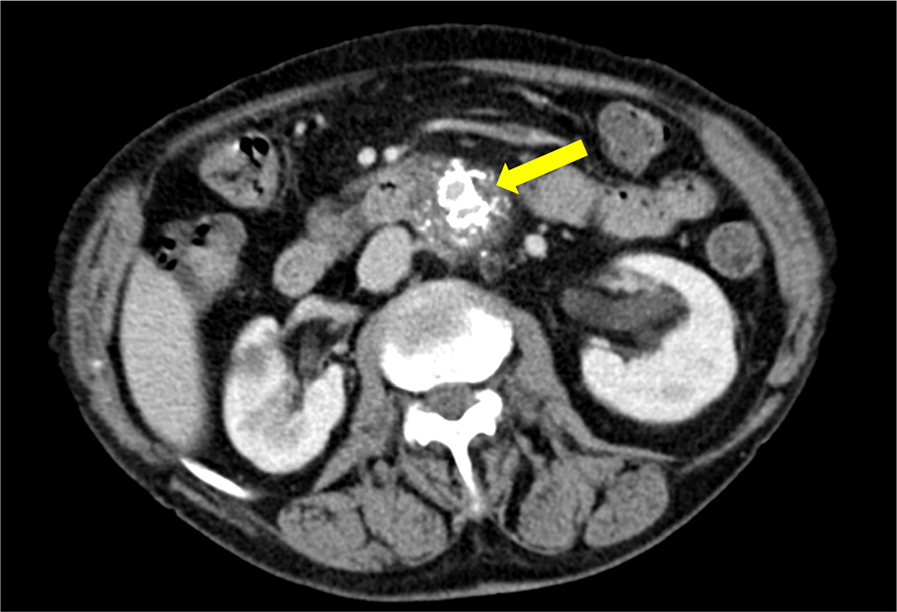
**CT-angiography control at 6 months.** The arrow indicates the slight narrowing of the iliac legs of the graft.

The mean follow-up period was 10 months (range 1–22 months). No deaths or major complications were reported during follow-up. No type I, III, or IV endoleaks, AAA enlargement, AAA rupture, stent fracture, migration, or secondary endovascular procedure, or conversion to open surgery were reported during the follow-up period. An asymptomatic narrowing of both iliac limbs was observed in one patient at 6 months (Figure 2), which is still on follow-up. The patient is taking oral anticoagulant for a permanent atrial fibrillation. There was a type II endoleak from lumbar arteries in two patients who are still under surveillance. Neither the former nor the latter complications occurred in both patients who had received the graft using the “Chimney” technique.

## Discussion

Since Parodi’s initial experience [1], the need to have appropriate vascular access to deliver the aortic stent graft in EVAR has emerged as a main issue. As the endovascular experience grew, it soon became clear that when endovascular therapy was indicated for clinical criteria, it was often not feasible for anatomical reasons. The introduction of self-expandable materials permitted an extension of the method even in those patients who presented with small iliac-femoral access. Nevertheless, in the common practice, access site related complications are still observed, such as dissections, arterial ruptures and haematomas in case of percutaneous approaches. Reduced access site complications and lower overall procedural mortality rates after EVAR have been recently reported with the use of new lower-profile endograft systems [2-5].

The benefits of lower-profile devices are obvious. Not only is the risk of access site complications lower with smaller profile systems, but more patients with smaller access vessels can also be treated. However the downside of reducing the profile is the possible loss of proximal sealing, which can compromise the overall performance and durability of the device. For this purpose, not only were different anchoring systems introduced, but also prostheses with large free-flow. The theoretical assumption concerned the axiom that good sealing without migration meant absence of endoleak. It was realized that this axiom was true in part and that even in presence of graft migration sometimes there were no endoleaks.

The Ovation stent addresses these dual problems by combining a large free-flow with hooks with an inflatable ring begins 10 mm below the top of the covered part of the graft. The graft is fixed and the ring provides sealing to the arterial wall. By eliminating the need for a metallic endoframe entirely, the Ovation can keep a lower profile while achieving a good proximal sealing and likely a good durability over time.

In this sense our results are consistent with the purpose of the endograft since in the long-term we have not seen neither type I endoleaks nor graft migrations.

In our series, we observed some intraprocedural complications, all of which were successfully corrected with either a graft extension or fem-fem bypass surgery. On one hand, the reduction of the profile allowed the treatment of cases that would have been difficult or impractical to treat with other devices. On the other side, the reduction of the prosthesic structure might have increased the intraprocedural difficulties and also made cannulation of the gate more difficult, which in a patient of our case series led to a fem-fem bypass surgery.

**Figure 3:**
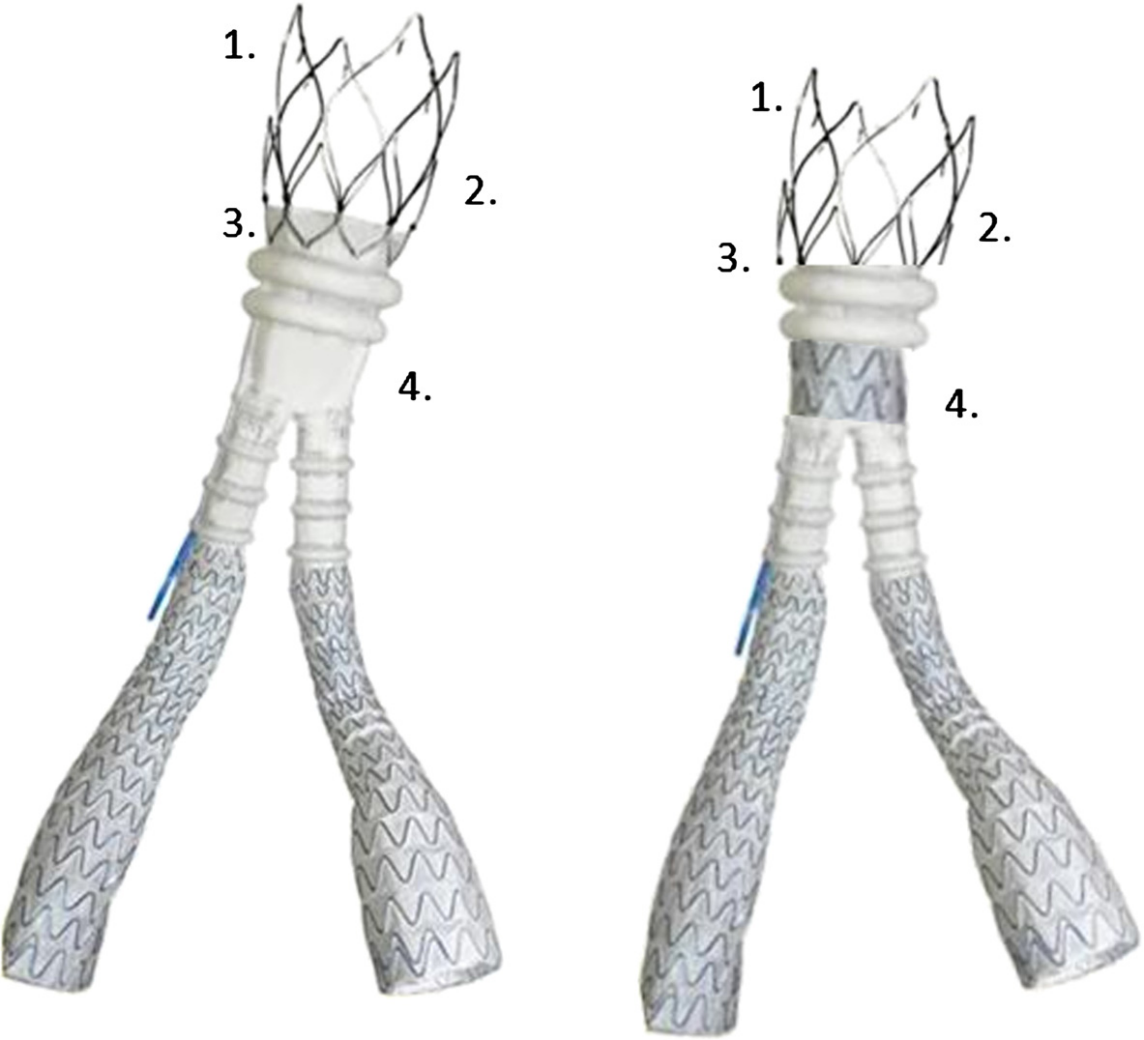
How to improve the Ovation Trivascular graft: 1. shorter free-flow; 2. less stiff free-flow; 3. shorter sealing collar above the ring; 4. strengthened distal part of the main body.

Notably, in our study, this was evident especially when proximal neck diameter was small. We could not explain the reason for this finding, as the complications observed usually are not correlated to proximal neck diameter. Moreover, the retrospective nature of our analysis and the small number of cases limit the strength of this conclusion. However, strengthening the distal part of the main body would probably be useful to facilitate cannulation of the gate and to avoid the shrinkage of the iliac legs, as observed in a patient of our study. Moreover, as the real proximal sealing is obtained through the polymer, the prosthetic ring above the sealing collar could be eliminated, improving the landing zone of the free-flow which could be shortened and made less stiff (Figure 3).

Speculating on graft modifications is beyond the scope of our study, however industries should address improvements of the graft considering real life experiences.

To some degree, the problems associated with cannulation have been addressed by the Ovation PRIME, the newest iteration of the endoprosthesis, which received CE Mark in June, 2012. In this version of the device, the contralateral leg is connected to the delivery system via a trigger wire, which is designed to prevent the twisting that caused the failure to cannulate described previously.

Albeit the experience is only at the beginning, the results reported in our series are encouraging, consistent with those reported in the Ovation pre-market clinical trial [6] in terms of aneurysm exclusion and prevention of aortic rupture.

Metha et al. [7] recently published a prospective, multicenter, single-arm trial to evaluate the one-year results about safety and effectiveness of the Ovation stent graft in 161 patients with AAAs. They reported a 30-day major adverse event rate of 2.5%, while at 1 year, AAA-related and all-cause mortality were 0.6% and 2.5%, respectively. Similarly to our results, no stent graft migration, no AAA rupture or type I, III, or IV endoleaks were reported in the long term but type II endoleaks were identified in 34% of patients and AAA-related secondary procedures were performed in 10 patients (6.2%) for 6 endoleaks, 3 aortic main body stenosis and 3 iliac limb stenosis or occlusion.

Similar outcomes are expected in UE post-marketing study. Moreover, the ongoing prospective non-randomized multi-center pre-market clinical trial [8] will probably build further understanding on the use of this device and its durability over time.

## Conclusions

Our initial experience with the Ovation endograft demonstrated encouraging early and long-term results in patients with AAA in terms of safety and effectiveness.

## Abbreviations

AAA: Abdominal aortic aneurysms; PTFE: Polytetrafluorethylene; PEG: Polyethylene glycol; FEP: Fluorinated ethylene propylene; ASA: Acetylsalicylic acid; OD: Outer diameter; CT: Computed tomography; SD: Standard deviation; ICU: Intensive care unit; OR: Odds ratio; CI: Confidence interval; EVAR: Endovascular aortic repair.

## Competing interests

The authors declare that they have no competing interests.

## Authors’ contributions

GN and DM participated equally, sharing "first authorship" for conception and design, data collection, writing the article and statistical analysis; SS, MTO, GM, DGT and AA participated in data collection, analysis and interpretation of the results; PV participated in conception and design, data collection and critical revision of the article. All authors read and approved the final manuscripts.

## References

[B1] Parodi JC Palmaz JC Barone HD Transfemoral intraluminal graft implantation for abdominal aortic aneurysms Ann Vasc Surg 1991 9 491 499 10.1007/BF02015271 1837729

[B2] Verhoeven BA Waasdorp EJ Gorrepati ML van Herwaarden JA Vos JA Wille J Moll FL Zarins CK de Vries JP Long-term results of Talent endografts for endovascular abdominal aortic aneurysm repair J Vasc Surg 2011 9 293 298 10.1016/j.jvs.2010.08.078 21055897

[B3] Bersin RM The Evolution of Low-Profile Endograft Design 2011 Evar Update: Endovascular Today 54 58

[B4] Moulakakis KG Dalainas I Kakisis J Giannakopolous TG Liapis CD Current knowledge on EVAR with the ultra-low profile Ovation Abdominal Stent-Graft System J Cardiovasc Surg 2012 9 427 432 22854522

[B5] Criado FJ The EVAR Landscape in 2011 2011 Evar Update: Endovascular Today 40 44

[B6] Presentation at the LINC 2012 (Leipzig International Course) by Dr. Dierk Scheinert: Featured research: Ovation European prospective multi-center study – 1 year data 2012 Leipzig January 25–28, 2012. Accessed from http://www.leipzig-interventional-course.com/index.php?option=com_content&task=view&id=183&Itemid=286 on October 23rd

[B7] Mehta M Valdés FE Nolte T Mishkel GJ Jordan WD Gray B Eskandari MK Botti C A Pivotal Clinical Study to Evaluate the Safety and Effectiveness of the Ovation Abdominal Stent Graft System” Investigators. One-year outcomes from an international study of the Ovation Abdominal Stent Graft System for endovascular aneurysm repair J Vasc Surg 2014 9 1 65 73 e3 10.1016/j.jvs.2013.06.065 23978572

[B8] Clinical Study of the TriVascular Ovation Abdominal Stent Graft System From *http://clinicaltrials.gov/ct2/show/NCT01092117*. Accessed on October 23rd, 2012

